# Transcriptomic profiling of *Escherichia coli* K-12 in response to a compendium of stressors

**DOI:** 10.1038/s41598-022-12463-3

**Published:** 2022-05-24

**Authors:** Rama P. Bhatia, Hande A. Kirit, Alexander V. Predeus, Jonathan P. Bollback

**Affiliations:** 1 Institute of Infection, Veterinary, and Ecological Sciences, University of Liverpool, Liverpool, Merseyside UK; 2 Laboratories of Molecular Anthropology and Microbiome Research, Stephenson Research and Technology Center, University of Oklahoma, Norman, OK USA

**Keywords:** Computational biology and bioinformatics, Microbiology

## Abstract

Environmental perturbations impact multiple cellular traits, including gene expression. Bacteria respond to these stressful situations through complex gene interaction networks, thereby inducing stress tolerance and survival of cells. In this paper, we study the response mechanisms of *E. coli* when exposed to different environmental stressors via differential expression and co-expression analysis. Gene co-expression networks were generated and analyzed via Weighted Gene Co-expression Network Analysis (WGCNA). Based on the gene co-expression networks, genes with similar expression profiles were clustered into modules. The modules were analysed for identification of hub genes, enrichment of biological processes and transcription factors. In addition, we also studied the link between transcription factors and their differentially regulated targets to understand the regulatory mechanisms involved. These networks validate known gene interactions and provide new insights into genes mediating transcriptional regulation in specific stress environments, thus allowing for in silico hypothesis generation.

## Introduction

Regulation of gene expression is important to organisms, as it allows them to survive fluctuating and stressful environments without changes in gene content. Bacterial gene expression is regulated by two tightly coupled processes, transcription and translation, that allow bacteria to survive numerous environmental insults. Bacterial regulatory proteins controlling the flow of genetic information often respond to environment signals by fine-tuning their transcriptional network. Identifying how they respond to distinct challenges will elucidate common and distinct processes that underlie physiological adaptation to challenging environments.

*Escherichia coli* (*E. coli*) is found as commensal member of the mammalian gut microbiome, and, in the case of some pathotypes, as an enteric pathogen^1^, in addition to being omnipresent in soil, water and plants. It is a model organism for biological research due to its non-pathogenic properties, easy handling, and a wide nutritional palate. Non-pathogenic *E. coli*, like the pathogenic variants, respond to sublethal environmental triggers by means of a stress response. The stress response may enable survival in addition to increasing the virulence potential of the bacterium^2^. The role of these plastic stress responses in persistence and virulence highlight the importance of studying changes in gene expression in response to stress to understand disease.

In *E. coli*, a global stress regulatory response is largely mediated by the sigma factors and the Rel/Spo homolog (RSH) proteins (p)ppGpp^3,4^. As such, changes in gene expression as a response to a stressful environment can lead to stress-induced cross protection to multiple other stressors. For instance, the stress response elicited by encountering carbon or nitrogen starvation also provides protection from high temperature, and otherwise lethal dose of hydrogen peroxide and disinfectants^5^. Similarly, studies have reported that an acid tolerance response in *E. coli* leads to the induction of heat shock proteins to maintain homeostasis^6^.

Gene expression analysis using RNA sequencing (RNA Seq) has been used extensively to elucidate *E. coli* response mechanisms to a variety of growth conditions. To mention a few examples, a transcriptomic analysis studying the factors involved in survival of pathogenic *E. coli* O157: H7 from digestion by the protist *Tetrahymena* revealed an upregulation of oxidative stress response genes, notably among which are *ahPF* and *katG*, that play a role in scavenging reactive oxygen species as well as other genes belonging to multiple stress responses^7^. Another study using a combination of RNA Seq and RT-qPCR investigated the transcriptome of *E. coli* O26:H11 in response to subinhibitory concentration of ciprofloxacin and reported an overexpression of the SOS response, Type III Secretion System (T3SS) effectors and a downregulation of motility^8^.

In addition to gene expression analysis, the Weighted Gene Co-expression Network Analysis (WGCNA), is an extensively used data mining technique for the study of complex biological interactions. It allows us to identify co-expression patterns between genes in a particular condition^9^. Based on co-expression similarity, the genes are clustered into modules that are representatives of specific biological processes^10^. The centrality-lethality rule points out that deleting a hub protein is more likely to be lethal than deleting a non hub protein. This is due to the fact that since a hub protein has multiple interactions, there is a higher probability for these hubs to be involved in essential interactions and also account for essential genes in the cell^11,12^. Identification of highly connected protein nodes (hubs/ hub proteins) from the protein–protein interaction (PPI) network in each co-expressed module will help understand the regulatory network architecture of *E. coli* when grown in a specific growth condition.

Few studies have used WGCNA to study gene co-expression in non-pathogenic *E. coli*^13,14^. Bacterial gene expression is highly sensitive to shifts in environmental factors like temperature, oxygen, nutrients, etc. To better understand the physiological adaptation to environments, it is important to disentangle the complex response elicited by bacteria when encountering a combination of multiple stressors in a heterogeneous environment. To address this we have used a systematic approach by exposing *E. coli* to multiple stress conditions that it potentially encounters in nature. We have examined global gene expression in *E.* coli using both differential expression and network approaches (i.e., WGCNA). These results clarify co-regulated gene networks, and the transcription factors that control differential gene expression.

## Materials and methods

### Bacterial strains and growth conditions

Overnight cultures of *E. coli* K12 strain MG1655 grown in different environments were diluted 1:1000 and grown at 37 °C and 220 rpm. On reaching the desired OD_600_, growth was stopped by adding Qiagen RNA Protect Bacteria Reagent (cat no. 76506). The antibiotic concentrations used were determined by performing an minimum inhibitory concentration (MIC) experiment for chloramphenicol and trimethoprim, and a concentration that gave twice the doubling time compared to growth in control media was chosen. The growth conditions are described in detail in Table 1. For each growth condition, two biological replicates were grown, except for Rich M9_1, which had four. Different OD600 values account for the differential growth rates of *E. coli* in different conditions.

**Table 1:** Growth conditions representing stress encountered by *E. coli.*

Environmental traits	Growth characteristics
Rich M9_1	Growth in M9 medium to OD_600_ 0.12 (4 replicates, cultures grown in deep well plates)
Rich M9_2	Growth in M9 medium to OD_600_ 0.4
Poor M9	Growth in M9 medium to OD_600_ 0.12
pH5	Growth in rich M9 medium at pH 5 to OD_600_ 0.4
Trimethoprim (TMP)	Growth in rich M9 medium supplemented with trimethoprim 0.3 µg/mL to OD_600_ 0.4
Chloramphenicol (CAM)	Growth in rich M9 medium supplemented with chloramphenicol 1.2 µg/mL to OD_600_ 0.4
Low Oxygen (LOX)	Growth in rich M9 medium with an overlay of paraffin oil to OD_600_ 0.4
Lennox Broth (LB)	Growth in Lennox (low salt) broth to OD_600_ 0.4

Rich M9: 1 × M9 salts, 1% CAA, 0.4% glucose, 2 mM MgSO_4_, 0.1 mM CaCl_2_.

Poor M9: 1 × M9 salts, 0.4% glycerol, 2 mM MgSO_4_, 0.1 mM CaCl_2_, 1 μg/ml thiamine.

### Library preparation and sequencing

Total RNA was isolated using the Qiagen RNeasy Mini Kit (cat no. 74104) and checked for purity and intactness with a Agilent 2100 Bioanalyzer. The libraries were prepared with ribosomal RNA depletion (Ribo—Zero, NEB) and were sequenced on Illumina HiSeq2500-v4, SR100 mode at the VBCF NGS Unit (https://www.vbcf.ac.at), resulting in 10.7–13.8 million single-end 100 bp Illumina reads per sample.

### Mapping of sequenced reads

RNA-Seq data quality control, alignment, quantification, and statistics calculations were done using the “simple” workflow of Bacpipe RNA-seq processing pipeline v0.6.0 (https://github.com/apredeus/multi-bacpipe), using the reference genome and annotation of *E. coli* strain MG1655 from NCBI (genome sequence GenBank ID U00096.3; assembly ID GCA_000005845.2). Annotation of tRNA and rRNA features was performed independently using Prokka v1.13.3. Basic read quality control was performed with FastQC v0.11.8. Reads were aligned to the genome sequence using STAR v2.6.0c using the “--alignIntronMin 20 --alignIntronMax 19 --outFilterMultimapNmax 20” options. For 20 processed samples, 96.2–97.9% reads were mapped successfully; 0.6–2.4% of these mapped to rRNA operons and were removed from further analyses. The remaining 92.0–95.5% of reads mapped to the rest of the genome, with 1.0–1.5% mapped to more than 1 location. Overall, 10.0–13.1 M reads aligned to the genome uniquely for each sample; another 0.13–0.19 M reads aligned non-uniquely to non-rRNA regions.

For RNA-seq quantification, a processed GFF file was generated by Bacpipe, where all features of interest were listed as a “gene”, with each gene identified by MG1655 locus tag. Resulting GFF file contained 4566 features (4240 protein coding, 147 pseudogene, 71 noncoding RNA, 22 rRNA, and 86 tRNA). Following this, read counting was done by featureCounts v1.6.4, using options “-O -M --fraction -t gene -g ID -s 2”, since the library was sequenced using a dUTP-based strand-specific protocol (Supplementary Table). Overall, 95.0–97.6% of initial reads were assigned to an annotated feature.

For visualization, scaled gedGraph files were generated using bedtools genomecov with a scaling coefficient of 109/(number of aligned bases), separately for sense and antisense DNA strands. Bedgraph files were converted to bigWig using bedGraphToBigWig utility (Kent utilities, http://hgdownload.soe.ucsc.edu/admin/exe/linux.x86_64/). Coverage tracks, annotation, and genome sequence were visualized using JBrowse v1.16.6, and are available at the following URL: http://hintonlab.com/jbrowse/index.html?data=MG1655/data.

## Analysis

### Differential gene expression

Differentially expressed genes (DEGs) were identified by subjecting raw counts to DeSeq2 package (Love et al., 2014) in R version 4.0.2. The Benjamini and Hochberg False Discovery Rate (FDR) criterion proposed by (Reiner-Benaim, 2007) was used to compute *P*_*adj*_ values. An absolute value of log_2_ fold change > 2 (i.e., a fourfold difference in either direction) and an *P*_*adj*_ < 0.001 was used as the threshold for selecting DEGs.

### Stress response network (SRN)

Genes involved in *E. coli* K12 stress response were obtained using GO term GO:0006950 (Response to stress) from Ecocyc^15^. Protein–protein interaction (PPI) networks were constructed for (i) genes in GO:0006950 and (ii) DEGs identified in *E.* coli K12 grown in carbon and amino acid starvation, low oxygen, presence of antibiotic stress and low pH, using the Search Tool for the Retrieval of Interacting Genes/Proteins (STRING) database version 11.0b using high confidence (cutoff score: 0.7). Each of these networks were exported to Cytoscape 3.8.0 and a union of the two networks was created to be used as the final Stress Response Network (SRN). Densely connected regions in the network representing important pathways were identified using MCODE (Molecular Complex Detection) clustering algorithm^16^. Additionally, the network was analysed to identify crucial stress response proteins. Functional enrichment of Gene Ontology (GO) annotations was performed using Database for Annotation, Visualization, and Integrated Discovery (DAVID) 6.8 and was visualized using MonaGO^17^.

### Co-expression network analysis

Signed weighted gene co-expression networks were constructed for the dataset using the Weighted Co-Expression Network Analysis (WGCNA) package^9^ in R version 4.0.2. Firstly, to reduce the effect of noise due to low expression, genes where the sum of counts across all samples was < 10 TPM (Transcripts Per Million) were discarded from further analysis^18^ (297 genes were discarded). A variance stabilizing transformation was then applied to the TPM counts using the DESeq2 package (Love et al., 2014). The goodSamplesGenes function was used to ensure the dataset had no missing values. A distance matrix was created for the samples and hierarchical clustering was applied to detect any sample outliers. Hierarchical clustering on the distance matrix of samples did not detect any outliers, hence all samples were retained for the analysis. Network topology analysis was performed using multiple soft-thresholding powers to obtain reliable scale independence and mean connectivity measures. Based on a scale-free topology criterion, an appropriate soft-thresholding power b was chosen using the pickSoftThreshold function. We chose the power for which the scale-free topology fit index (R^2^) was > 0.80. The Pearson’s correlations were raised to a power (b) of 14 to create a weighted adjacency matrix, which was then transformed into a Topological Overlap Matrix (TOM) and the corresponding dissimilarity was calculated to reduce the effects of pseudo associations. The topological overlap for a pair of genes is calculated by comparing their connections with all other genes in the network. Genes sharing the same neighbourhood are said to have a high topological overlap^19^. The TOM matrix was used as an input to create a dendrogram of genes using average linkage hierarchical clustering. Each leaf in the dendrogram represents a gene and the highly interconnected and co-expressed genes are grouped together by the branches. Gene modules were identified by cutting the branches off the dendrogram using the cutreeDynamic function and using a minimum cluster size of 30 genes. Modules with similar expression profiles were merged by clustering the module eigengenes and using the mergeCloseModules function with a height cut of 0.25. Eigengenes of the new merged modules were calculated and each module was identified with a colour, grey colour representing a module of uncorrelated genes. Module eigengenes were correlated to the environmental traits in the dataset to look for the most significant associations. P-values were calculated for the correlations and an FDR correction was applied to compute *P*_*adj*_ values. Significant modules were identified using a cut-off criteria of correlation value > 0.7 and *P*_*adj*_ ≤ 0.005.

### Network construction and identifying hubs

Protein–protein interaction (PPI) networks were constructed for the significant modules using the Search Tool for the Retrieval of Interacting Genes/Proteins (STRING) database version 11.0b with default parameters. The interactions from the STRING database were exported to Cytoscape 3.8.0 and the top 10 protein hubs for each module were identified using the Maximal Clique Centrality (MCC) algorithm of the CytoHubba plugin. The MCC algorithm was chosen for hub identification, as it performs better in comparison with the other algorithms of the CytoHubba plugin and can capture both high and low degree essential proteins in a network^20^.

### Identification and distribution of transcription factors (TFs) and sigma factors

A collection of TFs and sigma factors was obtained using the RegulonDB version 10.8 datasets supported by experimental evidence. In order to identify key genes induced by stress, we analysed the DEGs in each of the stress environments in our study to look for TFs and their regulated gene targets.

An enrichment analysis was also carried out using the hypergeometric test in R version 4.0.2 to identify modules highly enriched in TFs and sigma factors and comprehend their association with the co-expression modules.

### Differentially co-expressed genes (DCGs)

The WGCNA function in the DCGL package in R version 4.0.2 was used to identify DCGs for relevant pairwise comparisons of different environments.

Alternatively, overlapping genes between the co-expressed genes in the hub modules and the respective DEG set were also identified. Briefly, for environmental traits with more than one significant module, co-expressed genes were pooled into a single list and the genes overlapping with the DEGs were identified. They are referred to as differential co-expression overlap gene set (DCOGs) in this paper. TFs from DCOGs and the gene targets regulated by them were identified and the targets showing differential regulation were extracted for further study.

### Functional enrichment analysis

Gene Ontology Biological Processes (GO-BP) and Kyoto Encyclopaedia of Genes and Genomes (KEGG) Pathway enrichment analysis were carried out using the ClusterProfiler package in R^21^ to determine the processes and pathways regulated by the co-expression modules, transcription factors and their DE targets. Enriched terms were identified using a cut-off criteria of *P*_*adj*_ ≤ 0.01.

## Results

### Identification of differentially expressed genes (DEGs)

**Figure 1:**
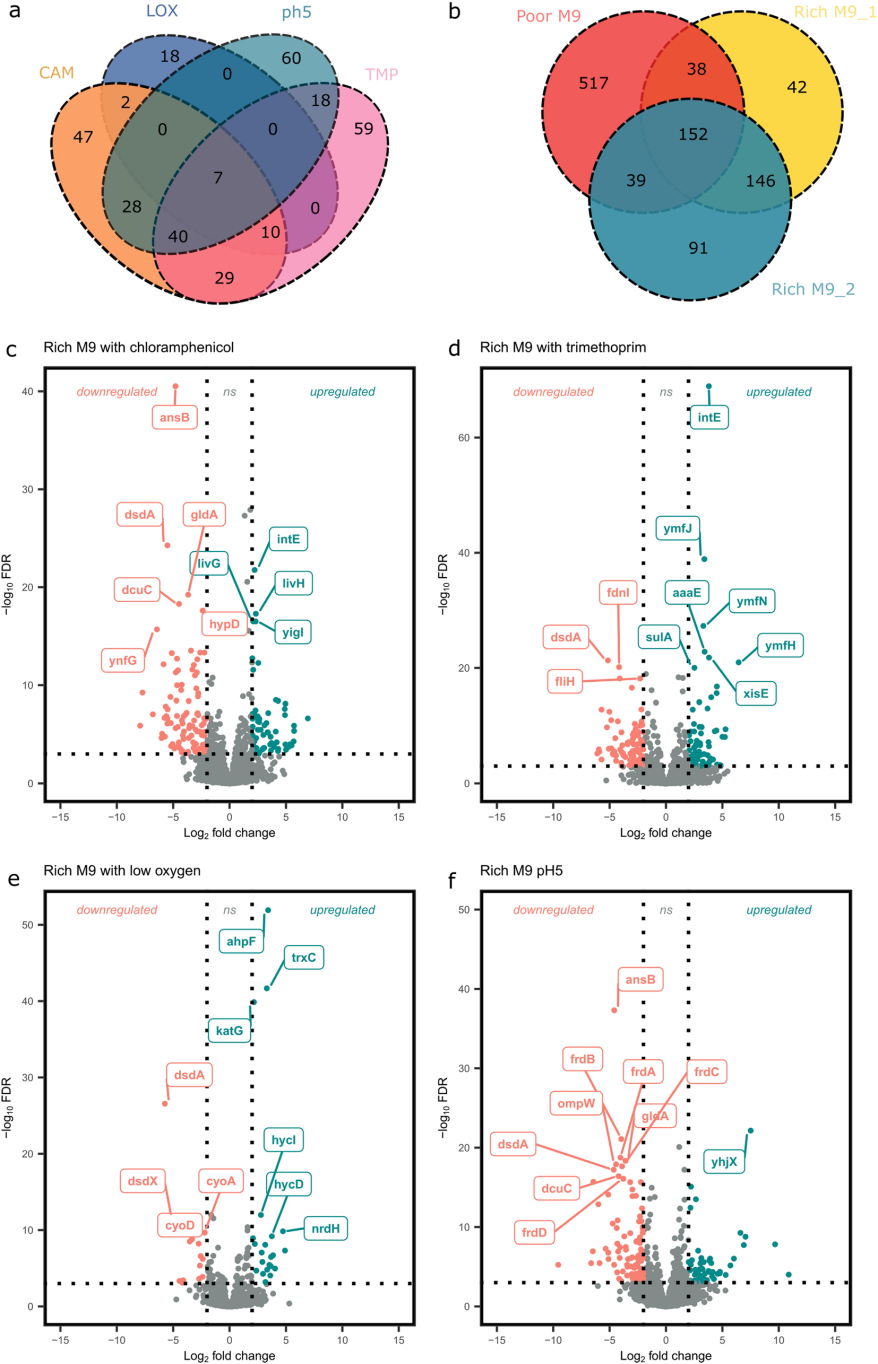
Differential expression in different environments. Venn diagrams depicting the number of genes showing significant differential regulation (> 4-fold) in stress environments in comparison to Rich M9 (**a**) and in nutrient limiting conditions compared to Lennox Broth(LB) (**b**). Volcano plots showing the top 10 differentially expressed genes for *E.coli* K12 grown in Rich M9 with 1.2 µg/mL chloramphenicol (**c**), 0.3 µg/mL trimethoprim (**d**), low oxygen (**e**) and pH5 (**f**), each in comparison with Rich M9. The vertical dotted lines are indicative of the log_2_ fold change thresholds and the horizontal dotted line depicts an FDR threshold (*P*_*adj*_ < 0.001).

Transcriptomic changes were studied by identifying DEGs showing significant fourfold difference in gene expression in either direction. To understand the effect of environmental stress on gene regulation, we compared antimicrobial (Fig. 1a,c,d), acidic pH (Fig. 1a,f), and low oxygen environments (Fig. 1a,e) to the Rich M9 environment. The effect of nutrient limitation was analysed by comparing gene expression data in minimal environments (Rich M9 and poor M9) to a nutrient rich growth media (LB) (Fig. 1b). In comparison with the nutritionally rich growth condition, growth in poor M9 showed the highest number of DEGs, a total of 746 genes were identified, of which 558 genes were significantly up-regulated and 188 genes were significantly down-regulated. In comparison with Rich M9, growth in antimicrobial containing environments had the highest number of DEGs.

### SRN network

The SRN generated consisted of 1431 nodes and 12,076 edges. Topological analysis of the network using the NetworkAnalyzer plugin in Cytoscape 3.8.0 showed that the highest degree was 157 and the average was 18.184. Degree of a node represents the number of nodes connected to it. Nodes with a higher degree are likely to be considered as hubs or central proteins in the network^22^. Using degree of a node as a measure of centrality, we identified 33 central proteins. We hypothesize that these proteins are crucial in responses to a variety of stressors and might also be involved in mediating stress-induced cross-protection. The list of proteins central to stress response and their literature annotations can be found as Supplementary Table S1 online. Out of the 33 central proteins identified, 24 were found to have known roles in stress response.

**Figure 2:**
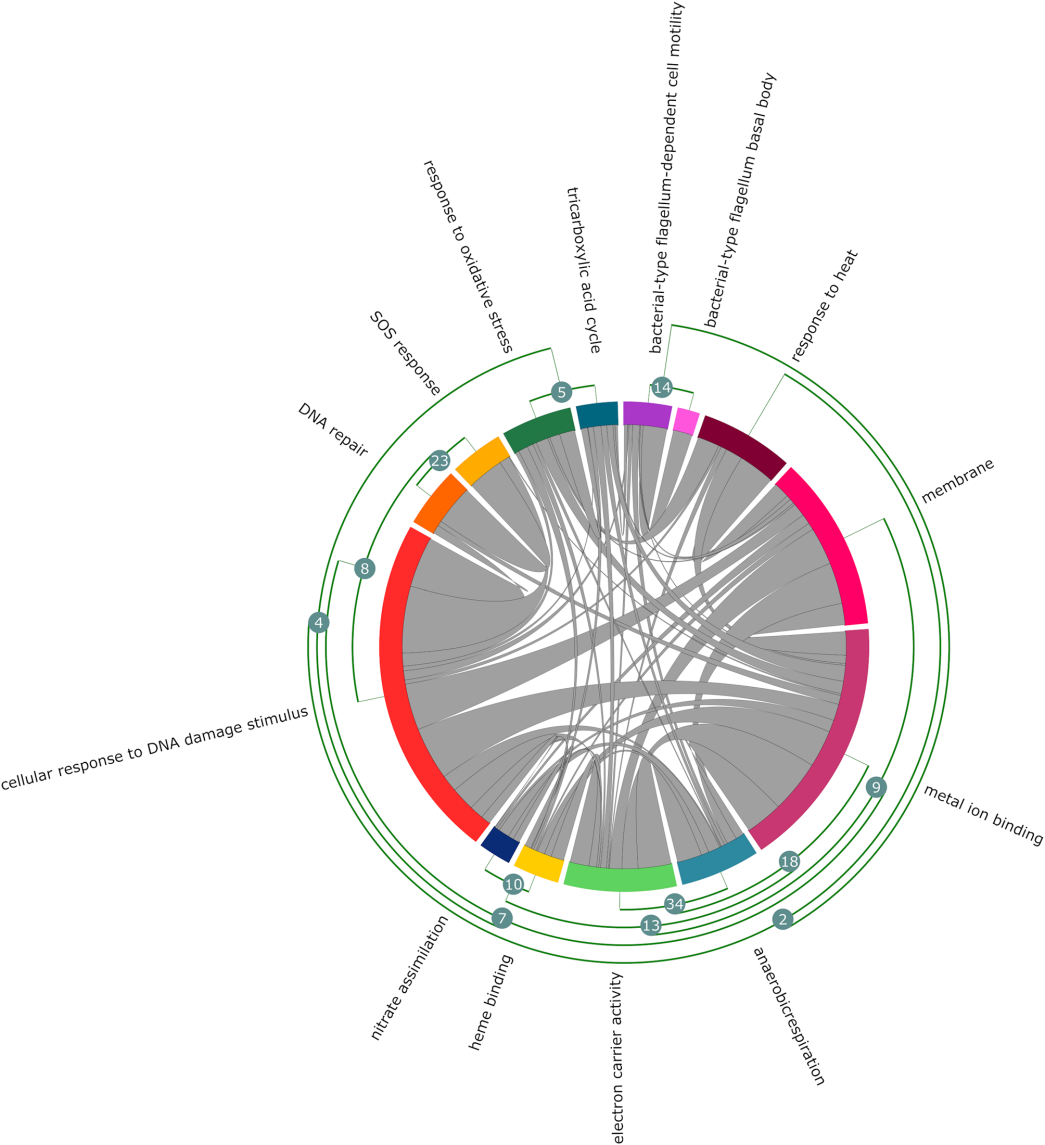
Cross-talk between significantly enriched GO annotations in SRN. Chord diagram depicting the connections between significantly enriched GO annotations (*P*_*adj*_ < 0.01). The fragments on the outer ring of the circular layout represent the nodes/GO annotations. The nodes are clustered based on the number of overlapping genes. The curved arcs within the circle show the interactions between different annotations.

Five sub-networks using the DEGs were generated for each of the stress environments—Poor M9, CAM, TMP, LOX, and pH5. We observed that a majority (> 70–80%) of these central proteins were present in each of the stressor specific sub-networks, except for the LOX sub-network which had 40% of the central proteins present. The genes in top five densely connected clusters in the SRN identified by MCODE belonged to Flagellar assembly, Energy metabolism, SOS response and DNA Repair, RNA binding proteins, and Biosynthesis of amino acids and secondary metabolites. Genes involved in each of the clusters are outlined in Supplementary Table S2 online. Functional enrichment of SRN identified response to heat and oxidative stress, DNA repair, SOS response, TCA cycle, anaerobic respiration, nitrate assimilation, flagellum based cell motility and cellular response to DNA damage as significantly enriched (*P*_*adj*_ < 0.01) (Fig. 2).

### Co-expression networks

Signed networks were constructed to identify genes that are co-expressed in the tested growth conditions as they take into consideration the sign of the correlation coefficients and can identify modules that are significantly positively or negatively correlated with the categorical variables or experimental conditions.

There are trade-offs for maximising R^2^ and retaining the number of mean connections, hence a power (β) of 14 was chosen.The network analysis identified a total of 20 co-expression modules and were assigned a colour each indicated at the bottom of the dendrogram.

On introducing environmental traits in the network, significant associations (modules) were identified using a threshold of correlation value (between module eigengenes (ME) and traits) > 0.7 and *P*_*adj*_ ≤ 0.005. Of the 20, 7 modules were identified with significant associations (Fig. 2). No modules were found to be significantly associated with Rich M9, Rich M9 supplemented with 1.2 µg/ml chloramphenicol (CAM) and Rich M9 acidic condition (pH5). Summary about the modules is given in Table 2. Detailed information about the genes in the modules can be found in Supplementary Table S3 online.

**Table 2:** Module summary.

Growth condition	Module	Correlation	p-adjusted	Gene count
Lennox Broth	Green	0.77	0.002	472
	Dark Orange	−0.77	0.002	230
Poor M9	Blue	0.97	8e−11	691
	Dark Red	−0.76	0.002	503
	Purple	0.76	0.002	149
TMP	Orange	0.83	3e−04	61
LOX	Saddle brown	0.97	3e−10	41

**Figure 3:**
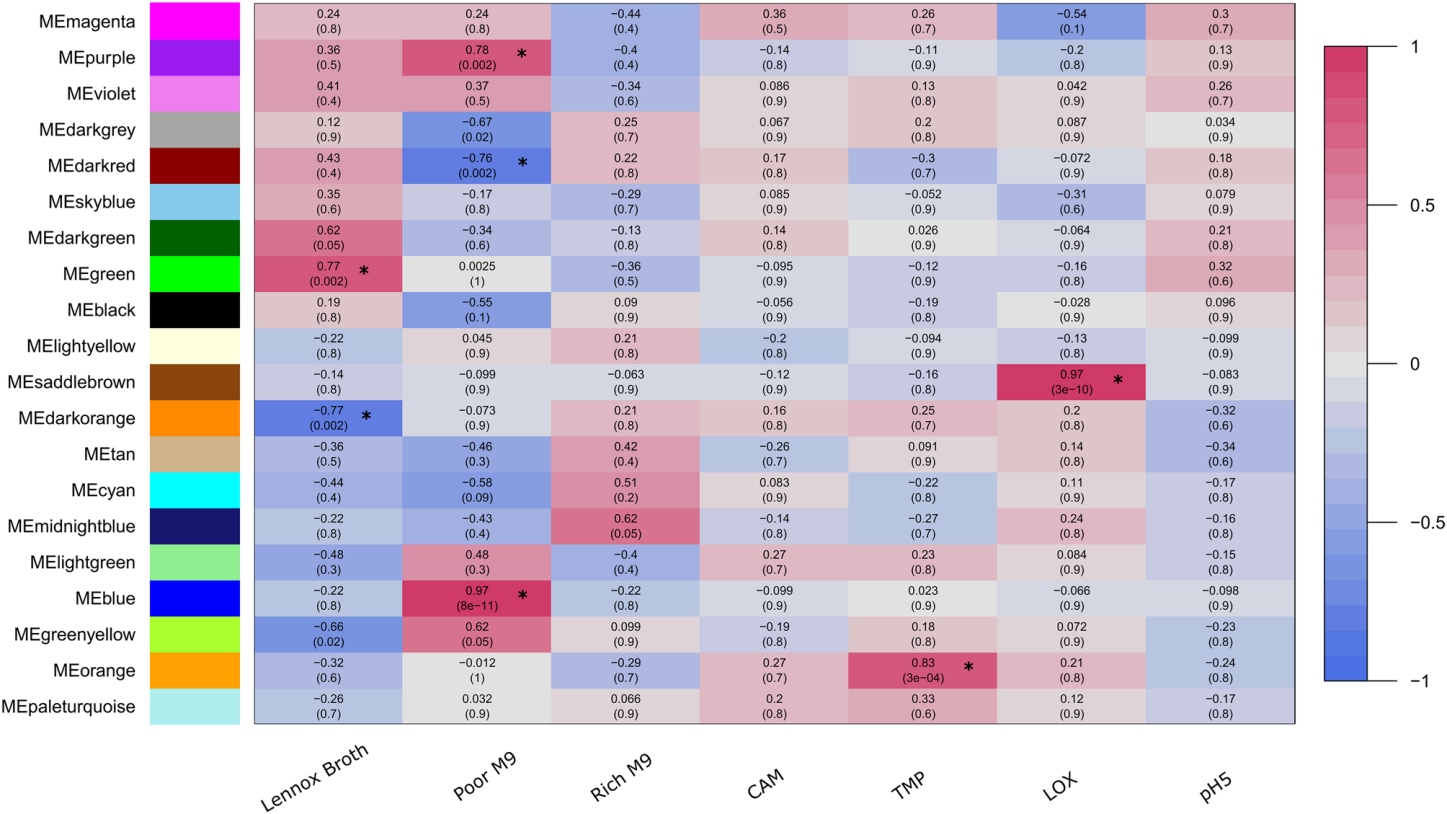
Module-trait relationships. Associations between module eigengenes (ME) and experimental conditions. Rows represent MEs and columns represent growth environments. Numbers outside and within parentheses are correlation coefficients and FDR corrected p-values of the correlations, respectively. Positive correlations are indicated by pink colour and negative correlations by blue colour. Significant associations are highlighted with an asterisk sign (*).

**Figure 4:**
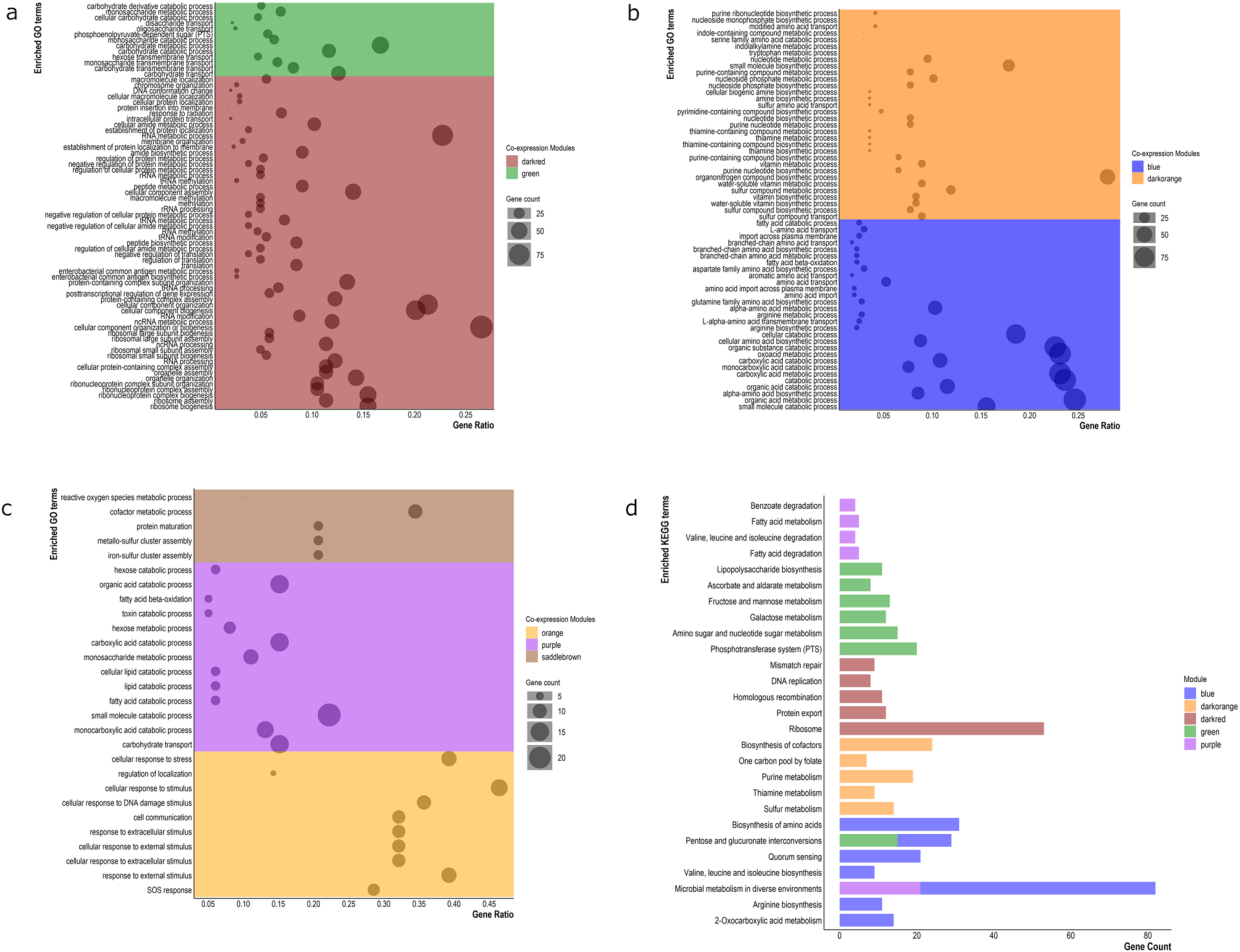
Functional and pathway enrichment analysis. Enriched GO-BP and KEGG terms in individual hub modules (*P*_*adj*_ < 0.01). The colours are representative of modules.

Functional enrichment analysis was carried out to understand the relationship between the modular biological functions and the experimental conditions. Analysis of *E. coli* K-12 grown in nutritionally rich Lennox broth identified two modules, green and darkorange (Fig. 3) that are positively and negatively correlated to the environment, respectively. The genes in the green module are significantly enriched in biological processes like carbohydrate metabolism and transport (Fig. 4a), whereas the darkorange module is involved in vitamin, amino acid, and nucleotide metabolic and biosynthetic processes (Fig. 4b). Three modules, blue, darkred and purple (Fig. 3) were found to be significantly associated with the minimal growth media—Poor M9. Of these, the darkred module (Fig. 4a) was significantly negatively correlated with the growth environment. Some of the GO-BP terms associated with the darkred module include ribosome biogenesis, RNA metabolic processes, post-transcriptional regulation of gene expression, translation. The blue module was largely involved in organic substance catabolic processes and alpha-amino acid biosynthesis and metabolism(Fig. 4b). The purple module was mainly linked with small molecule catabolic process and carbohydrate transport (Fig. 4c). Growth of *E. coli* K-12 in Rich M9 medium supplemented with trimethoprim 0.3 µg/mL was found to be significantly associated with genes in the orange module (Fig. 3). The orange module is associated with processes like SOS response, cellular response to DNA damage, stress, and external stimulus (Fig. 4c). Genes in the saddle brown module were positively correlated with the low oxygen (LOX) environment (Fig. 3) and were majorly involved in cofactor metabolic processes, iron-sulfur cluster assembly and protein maturation (Fig. 4c). In the KEGG enrichment analysis, 27 pathways were significantly enriched among five of these modules, some of which include microbial metabolism in diverse environments, quorum sensing and phosphotransferase system (PTS) (Fig. 4d). No significantly enriched KEGG terms were found to be associated with the orange and saddle brown module.

### Protein–protein interaction (PPI) networks and screening hub genes

**Figure 5:**
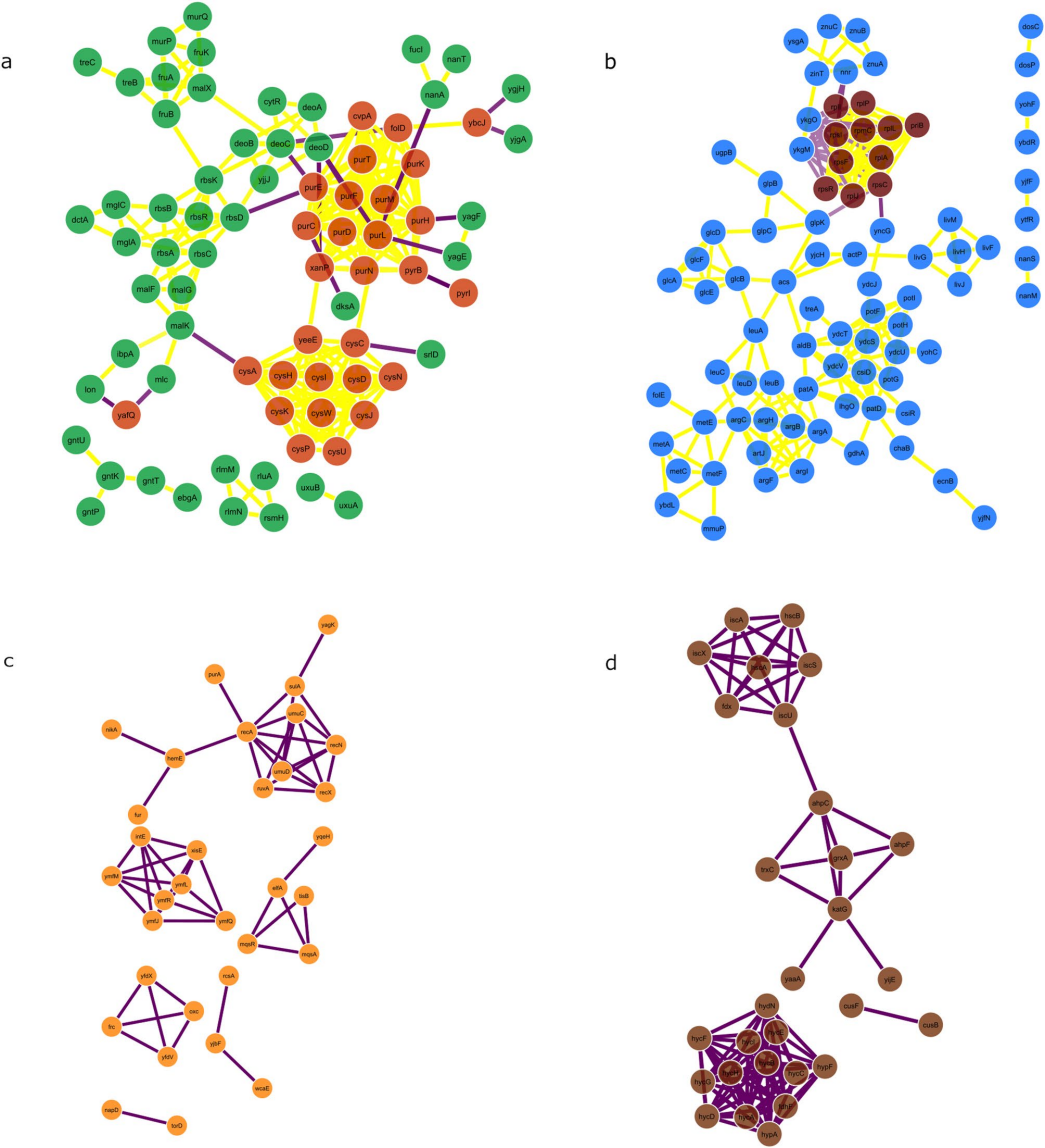
PPI networks. Protein–protein interactions of gene products for significant module-environment associations identified. Modules were subsetted based on their intramodular connectivity on a scale of 0 to 1. Subset of highly interconnected genes in LB (**a**), Poor M9 (**b**), Rich M9 with 0.3 µg/mL trimethoprim (**c**) and Rich M9—low oxygen growth (**d**). Due to the small number of PPIs for orange (trimethoprim) and saddle brown (low oxygen), these modules were not subsetted, Node colours represent module colours. Magenta and yellow edges show positive (both genes connected are either upregulated or downregulated) and negative (one gene is upregulated and the other is downregulated) correlation between genes, respectively.

PPI network interactions for the hub modules were obtained from the STRING tool and exported to Cytoscape for network visualization (Fig. 5). The MCC algorithm of the CytoHubba plugin in Cytoscape^20^ was used to identify the top 10 hub genes in each of the modules. Module hubs and their respective functions can be found as Supplementary Table S4 online.

### Distribution of TFs and sigma factors

**Figure 6:**
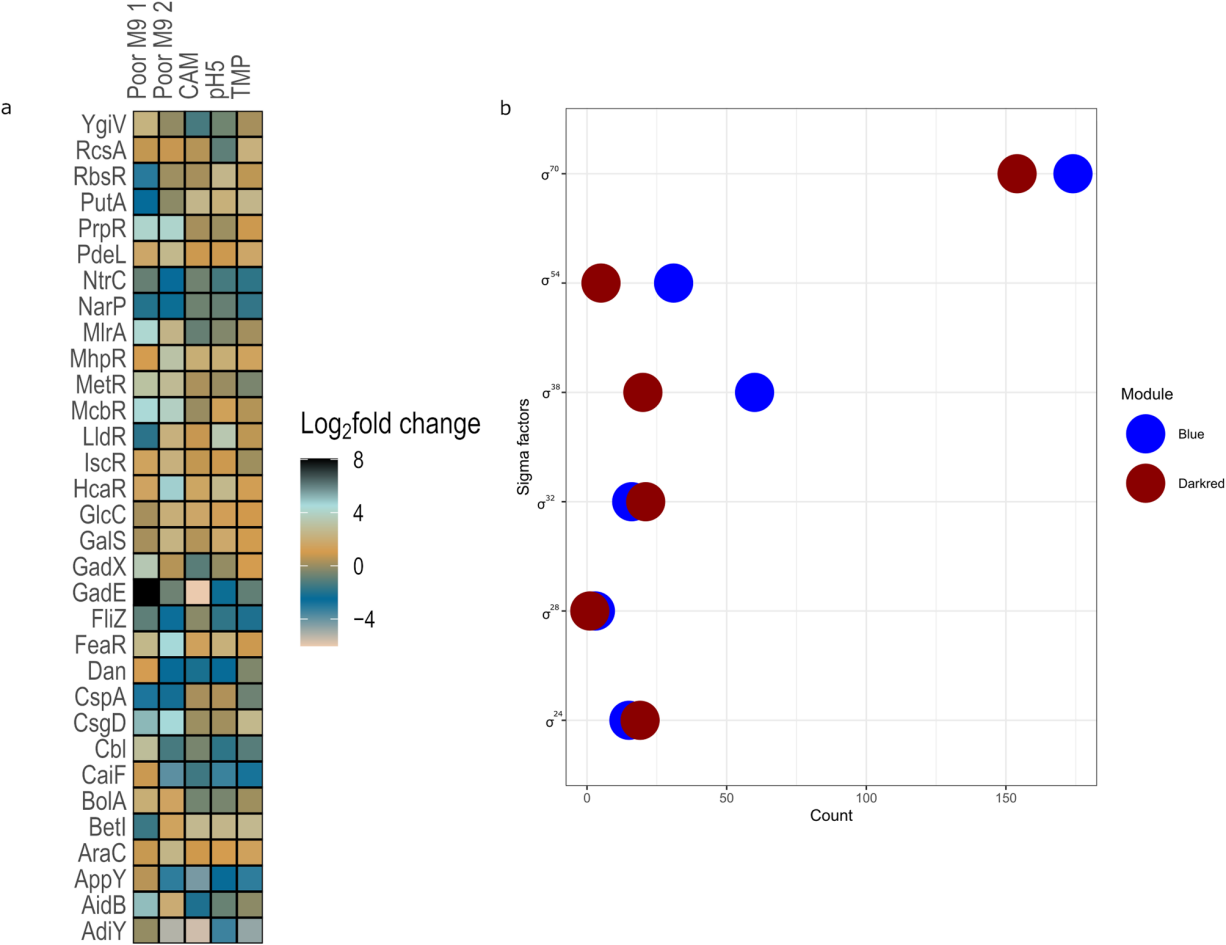
Distribution of transcription factors (TFs) and sigma factors. (**a**) Heatmap showing the log_2_ fold change value of the 32 differentially expressed TFs (from left to right) in Poor M9 1 compared with LB, Poor M9 2, CAM, pH5 and TMP compared with Rich M9. (**b**) Dot plot showing the distribution of sigma factors in blue and darkred modules.

Transcription factors rewire bacterial gene expression based on environmental signals allowing the bacterium to rapidly survive stress^23,24^. We found a total of 32 TFs to be differentially expressed across Poor M9 (individually compared to LB and Rich M9), CAM, TMP and pH5. The frequently occurring TF families found were AraC/Xyls, LuxR/UhpA and LysR. A comprehensive list for each stress environment detailing the TFs identified, their family classification, target genes and their differential expression status in that environment can be found in Supplementary Tables S5-S9 online. Figure 6a shows differential expression of the 32 TFs across Poor M9, CAM, TMP and pH5.

Additionally, the distribution of experimentally validated TFs and sigma factors in the modules was identified. Based on the enrichment analysis using the hypergeometric distribution, two modules, green (p-value = 0.001) and purple (p-value = 0.02) were found to be significantly enriched in TFs. To identify the pivotal pathways regulated by TFs during stress, a KEGG enrichment analysis was carried out for the differentially regulated TF -gene targets only in the purple module (significantly associated with carbon and nitrogen starvation environment). The KEGG analysis identified quorum sensing to be significantly enriched (*P*_*adj*_ < 0.01). Out of the seven modules, only two modules (blue and darkred—significantly associated with Poor M9 environment) were found to be significantly enriched (*P*_*adj*_ < 0.05) in sigma factors. Figure 6b shows the distribution of sigma factors in the blue and darkred modules. The entire list of sigma factor regulated genes in the two modules can be found as Supplementary Table S10 online.

### Association between differentially expressed and co-expressed genes

The WGCNA function in the DCGL package did not identify any differentially co-expressed genes in the tested pairwise comparisons.

To understand the link between differential expression and co-expression, a list of all genes that were significantly co-expressed in an environment were compared to the list of genes differentially expressed in the same environment. This analysis was carried out only for the carbon and amino acid starvation condition (Poor M9), low oxygen (LOX), and antimicrobial stress (TMP), as significant co-expression was observed only in these environments (Fig. 3). DCOGs or the genes commonly identified between differential expression and co-expression were extracted for each of the stress environments—Poor M9, LOX and TMP. The list of DCOGs was further analysed to look for the presence of TFs and its respective differentially regulated targets. A KEGG pathway analysis was carried out for the identified TFs and its differentially regulated targets, in an effort to understand the regulation of transcriptional network by bacteria when encountering stress. No TFs were identified in the DCOGs for the low oxygen (LOX) environment. Six TFs were identified in the DCOGs for Poor M9 and the flagellar assembly pathway was found to be significantly enriched (*P*_*adj*_ < 0.01) in KEGG analysis. A single TF RcsAB was identified in the DCOGs for environment containing the antibiotic trimethoprim (TMP) and the KEGG analysis revealed the biofilm formation pathway to be significantly associated (*P*_*adj*_ < 0.01).

## Discussion

In this study we use RNA-Seq and WGCNA to understand the functional modules and co-expression networks involved in the stress response of *E. coli* K12.

The stress response network (SRN) identified 33 proteins that are central to *E. coli* stress response. The presence of majority of these proteins in stressor specific sub-networks indicates the essentiality of these proteins in mediating bacterial response to a variety of stressors.

The Poor M9 medium is a representative of carbon limitation and amino acid starvation conditions. Analysis of TFs showed that genes involved in response to sulfate starvation, curli assembly and transport, biofilm formation, maintenance of pH homeostasis, arabinose catabolism, glycolate utilization were induced in response to starvation, indicative of their role in response to starvation. Based on co-expression analysis, nutrient depletion and amino acid stress activates a stringent response pathway in *E. coli*. The pathway is mediated by genes *relA* and *spoT* through the synthesis of the alarmone (p)ppGpp^25^. (p)ppGpp serves a global regulator for gene expression and redirects gene transcription from genes required for growth to genes required for survival during starvation^4^. The modules blue, darkred and purple were identified as being significantly associated with the Poor M9 medium (Fig. 3). The darkred module (Fig. 4a) was significantly negatively associated with the Poor M9 medium and was involved in translation, ribosome biogenesis, RNA metabolic processes and post-transcriptional regulation of gene expression, which is indicative of the induction of the stringent response pathway^26^. Although, the genes involved in mediating this pathway, *relA* and *spoT*, were not found to be differentially expressed in the Poor M9 medium in our dataset, previous studies have reported a decrease in basal levels of (p)ppGpp with high levels of SpoT^27,28^. Also, the “hopping model” for RelA mediated (p)ppGpp synthesis suggested that during a stringent response, RelA may hop between ribosomes, thereby allowing low enzyme concentrations to produce sufficient levels of (p)ppGpp^29^. However, this model has not been supported by other studies^30,31^ and the mechanistic details of (p)ppGpp synthesis via RelA remains unclear. Based on this, it can be inferred that the darkred module is involved in regulatory interactions leading to the activation of the stringent response pathway under starvation conditions. On the other hand, the blue (Fig. 4b) and purple (Fig. 4c) modules, found to be positively correlated with Poor M9, are mostly involved in amino acid biosynthesis and import, and catabolism. This can be attributed to the stringent response as well as the RpoS dependent stress response mechanisms. RpoS is known to be a master regulator of stress response, required for adapting to growth in conditions with glycerol as the sole carbon source by inducing the carbon scavenging mechanisms in the cell^32^. Out of the 558 genes significantly upregulated in the Poor M9 medium, 105 were found to be RpoS-dependent. Also, an increase in the (p)ppGpp level due to the activation of the stringent response pathway favours the transcription of s^38^ (RpoS) dependent promoters^33^. Several studies have shown that a rise in the alarmone ((p)ppGpp) levels, in co-ordination with the nutrient responsive transcription factor DksA, activate transcription of genes involved in de novo amino acid biosynthesis and import^34–37^. The RpoS dependent stress response also activates the transcription of *poxB* and *acs* (differentially upregulated in Poor M9) involved in carbon metabolism converting pyruvate to acetyl coenzyme A as well as other genes involved in catabolic reactions^33,38^.

This explains the role of the co-expressed genes identified in the three modules significantly associated with Poor M9. Furthermore, our analysis also shows how the (p)ppGpp mediated stringent response activated by coexpressed genes in the darkred module feeds into the induction of coexpressed genes in blue and purple modules, thereby activating the RpoS dependent stress response mechanisms. This explores a potential link between the three different coexpressed gene modules in an environment.

Based on the hypergeometric distribution, the purple module was significantly enriched in transcription factors. A KEGG analysis of the DEG regulated by the TFs revealed the quorum sensing pathway to be significantly enriched and the genes mediating the quorum sensing (QS) pathway belong to *lsr* operon. Although, a previous study showed that the presence of glycerol and glycerol-3-phosphate (G3P) repress the *lsr* operon^39^, the *lsr* operon genes are found to be significantly upregulated in our study, making it an interesting experimental candidate for further exploration of the role of *lsr* operon in nutrient limiting conditions. Also, it would be interesting to see if there is a link between the stringent response and the *lsr* mediated QS circuit in *E. coli* K-12. Links between the stringent response and QS regulation have been demonstrated in the enterohemorrhagic *E. coli* O157:H7 EDL933 strain^40^. In addition, the blue and darkred modules were found to be significantly enriched for sigma factors. Majority of the genes in these modules were under the control of σ^70^ (regulation of housekeeping genes), followed by σ^54^ (regulation of genes involved in nitrogen metabolism), σ^38^ (regulation of stationary phase genes), σ^32^ (heat shock regulating genes), σ^24^ (extreme heat shock genes) and a small number of genes under the control of σ^28^ (regulation of flagellar proteins)^41^. The data confirms that a starvation response offers cross-protection to high temperatures in *E.* coli, and is also supported by the GO enrichment analysis of SRN (Fig. 2).

Analysing the transcriptional regulation using the association between differential expression and co-expression indicated that the Poor M9 medium was enriched in flagellar assembly. Genes involved in the pathway were under the control of the CsgD transcription activator/master biofilm regulator, that is known to repress the flagellar assembly genes^42^. This is in line with the upregulation of the CsgD (Curli subunit gene D) cascade (genes involved in curli assembly and transport) and downregulation of the flagellar genes observed in our study. *E. coli* can switch between a sessile lifestyle regulated by CsgD and motile planktonic growth regulated by the flagellar cascade depending on environmental signals. Studies have reported a “foraging behaviour” in *E. coli* in response to poor carbon sources (e.g., glycerol, glycine, and succinate), in which bacteria activate the costly mechanism of flagellar synthesis in order to access better growth conditions^43–45^. Six non-coding, small RNAs (sRNA) (OmrA, OmrB, GcvB, RprA, McaS, ArcZ) have been reported to fine-tune the interplay between curli mediated biofilm state and flagellar mediated motility^46,47^. The expression levels of the six sRNAs were analysed and were indicative of curli mediated biofilm formation in Poor M9 in this study. Pathogenic *E. coli* may undergo rapid biofilm dispersal and revert back to single cell planktonic state^48^, suggesting that cell motility is related to virulence/pathogenicity, whereas biofilm formation is a mechanism of defence against stress^49^.

Trimethoprim (TPM) is an inhibitor of bacterial DNA synthesis by inhibiting dihydrofolate reductase, thereby preventing thymine incorporation^50^. Exposure to antibiotic stress lead to the upregulation of curli genes and genes involved in synthesis of colanic acid capsule (See Supplementary Table S8 online). WGCNA identified the orange module to be significantly associated with the TMP environment. The module is mostly involved in SOS response and cellular response to stimulus via *recA* expression (Fig. 4c), which confirms the facts known regarding SOS induction caused by trimethoprim. The SOS response is also known to induce filament production on exposure to trimethoprim by transcribing the SOS cell division inhibitor, *sulA*^50,51^, found to be differentially upregulated in the TMP environment in our study. The *rcsA* gene encoding the RcsAB transcription factor was identified in the DCOGs for TMP, and its differentially regulated targets (*csgDEF*) were enriched in biofilm formation. Bacteria are known to form biofilms as part of the SOS response, providing them with protection from antibiotic exposure and other harsh environments^42,52^. The Rcs system is a negative regulator of the *csg* operons in an Rcs-A dependent manner^53^, however, there is a significant upregulation of genes *csgD*, *csgE* and *csgF* of the *csgDEFG* operon as well as *csgB* of the *csgBA* operon in our study. The regulation of curli expression is a highly complex interaction, with more than ten transcription factors controlling the *csgD* promoter, each responding to a different aspect in stress related environments^54,55^. It will be interesting to explore if the antibiotic stress signal causes a derepression of the Rcs system to activate curli expression and biofilm formation.

The stress response generated by low oxygen environment was studied by sealing the *E. coli* cultures with a layer of paraffin oil, thereby limiting gas exchange. An air-saturated medium leads to endogenous production of hydrogen peroxide, initiating an oxidative stress response in *E. coli*^56^. Hydrogen peroxide signals the activation of the OxyR regulon, leading to the transcription of hydrogen peroxide resistance genes^57,58^, of which *katG*, *ahPF* and *trxC* were among the top 10 upregulated genes (Fig. 1e), *grxA* was significantly upregulated and *ahpC* was threefold upregulated in the LOX environment. Hydrogen peroxide destabilises bacterial iron-sulfur clusters and cause the release of molecular iron^59^. This explains the involvement of genes co-expressed in the saddle brown module significantly associated with LOX in the de novo assembly of iron-sulfur clusters via the *ics* operon (Fig. 4c). Although, hydrogen peroxide can inactivate the *ics* operon and induce the *suf* operon to compensate for the iron-sulfur cluster assembly, the effect is reversed with a decline in hydrogen peroxide stress^60^. Since, we did not see a strong expression of the *suf* operon genes in the LOX environment, combining the differential expression and co-expression analysis, it can be inferred that hydrogen peroxide is detoxified by the resistance genes, allowing for the *ics* operon to assemble and repair the damaged iron-sulfur clusters.

## Conclusions

In this study, we applied WGCNA on RNA-Seq data to identify relevant gene modules and the biological functions involved in *E. coli* stress response. Linking co-expression, differential expression and transcription factors allows us to find candidate genes that might help explore and further our understanding of the stress response cascade. In addition, further analysis of the module hubs might give useful insights into the regulation of co-expressed genes in a particular environment. Our data can lay the ground work for hypothesis based experimental validation of gene functions potentially involved in *E. coli* stress response mechanisms.

## Supplementary Information

Supplementary Table.

Supplementary Tables.

## References

[1] Kaper JB, Nataro JP, Mobley HL (2004). Pathogenic *Escherichia coli*. Nat. Rev. Microbiol..

[2] Chung HJ, Bang W, Drake MA (2006). Stress response of *Escherichia coli*. Comprehens. Rev. Food Sci. Food Saf..

[3] Boutte CC, Crosson S (2013). Bacterial lifestyle shapes stringent response activation. Trends Microbiol..

[4] Magnusson LU, Farewell A, Nystrom T (2005). ppGpp: A global regulator in *Escherichia coli*. Trends Microbiol..

[5] Matin A, Auger EA, Blum PH, Schultz JE (1989). Genetic basis of starvation survival in non-differentiating bacteria. Annu. Rev. Microbiol..

[6] Olson ER (1993). Influence of pH on bacterial gene expression. Mol. Microbiol..

[7] George AS, Rehfuss MYM, Parker CT, Brandl MT (2020). The transcriptome of *Escherichia coli* O157: H7 reveals a role for oxidative stress resistance in its survival from predation by Tetrahymena. FEMS Microbiol. Ecol..

[8] Valat C (2020). Overall changes in the transcriptome of *Escherichia coli* O26:H11 induced by a subinhibitory concentration of ciprofloxacin. J. Appl. Microbiol..

[9] Langfelder, P. & Horvath, S. WGCNA: An R package for weighted correlation network analysis. *BMC Bioinform*. (2008). 10.1186/1471-2105-9-559 PMC2631488 19114008

[10] van Dam S, Võsa U, van der Graaf A, Franke L, de Magalhães JP (2017). Gene co-expression analysis for functional classification and gene-disease predictions. Brief. Bioinform..

[11] Crua Asensio N, Munoz Giner E, de Groot NS, Torrent Burgas M (2017). Centrality in the host-pathogen interactome is associated with pathogen fitness during infection. Nat. Commun..

[12] He X, Zhang J (2006). Why do hubs tend to be essential in protein networks?. PLoS Genet..

[13] Liu W (2018). Construction and analysis of gene co-expression networks in *Escherichia coli*. Cells.

[14] Wang J (2013). Global metabolomic and network analysis of *Escherichia coli* responses to exogenous biofuels. J. Proteome Res..

[15] Keseler IM (2013). EcoCyc: Fusing model organism databases with systems biology. Nucleic Acids Res..

[16] Bader GD, Hogue CW (2003). An automated method for finding molecular complexes in large protein interaction networks. BMC Bioinform..

[17] Xin, Z. *et al.* MonaGO: A novel Gene Ontology enrichment analysis visualisation system. *bioRxiv*. (2020). 10.1186/s12859-022-04594-1 PMC8845231 35164667

[18] Kroger C (2013). An infection-relevant transcriptomic compendium for *Salmonella enterica* Serovar Typhimurium. Cell Host Microbe.

[19] Yip AM, Horvath S (2007). Gene network interconnectedness and the generalized topological overlap measure. BMC Bioinform..

[20] Chin C (2014). cytoHubba: Identifying hub objects and sub-networks from complex interactome. BMC Syst. Biol..

[21] Yu G, Wang L-G, Han Y, He Q-Y (2012). clusterProfiler: An R package for comparing biological themes among gene clusters. OMICS J. Integr. Biol..

[22] Fornito, A., Zalesky, A. & Bullmore, E. T. *Fundamentals of Brain Network Analysis* (eds. Fornito, A., Zalesky, A., & Bullmore, E.T.). 115–136. (Academic Press, 2016).

[23] Feugeas J-P (2016). Links between transcription, environmental adaptation and gene variability in *Escherichia coli*: Correlations between gene expression and gene variability reflect growth efficiencies. Mol. Biol. Evol..

[24] Guo MS, Gross CA (2014). Stress-induced remodeling of the bacterial proteome. Curr. Biol..

[25] Bullwinkle TJ, Ibba M (2016). Translation quality control is critical for bacterial responses to amino acid stress. Proc. Natl. Acad. Sci..

[26] Sanchez-Vazquez P, Dewey CN, Kitten N, Ross W, Gourse RL (2019). Genome-wide effects on *Escherichia coli* transcription from ppGpp binding to its two sites on RNA polymerase. Proc. Natl. Acad. Sci. U S A.

[27] Germain E (2019). YtfK activates the stringent response by triggering the alarmone synthetase SpoT in *Escherichia coli*. Nat. Commun..

[28] Murray DK, Bremer H (1996). Control of spoT-dependent ppGpp synthesis and degradation in *Escherichia coli*. J. Mol. Biol..

[29] Wendrich TM, Blaha G, Wilson DN, Marahiel MA, Nierhaus KH (2002). Dissection of the mechanism for the stringent factor RelA. Mol. Cell.

[30] Li W (2016). Effects of amino acid starvation on RelA diffusive behavior in live *Escherichia coli*. Mol. Microbiol..

[31] English BP (2011). Single-molecule investigations of the stringent response machinery in living bacterial cells. Proc. Natl. Acad. Sci. U S A.

[32] Martínez-Gómez K (2012). New insights into *Escherichia coli* metabolism: Carbon scavenging, acetate metabolism and carbon recycling responses during growth on glycerol. Microb. Cell Fact..

[33] Flores N (2007). New insights into the role of sigma factor RpoS as revealed in *Escherichia coli* strains lacking the phosphoenolpyruvate: Carbohydrate phosphotransferase system. J. Mol. Microbiol. Biotechnol..

[34] Roghanian M, Semsey S, Lobner-Olesen A, Jalalvand F (2019). (p)ppGpp-mediated stress response induced by defects in outer membrane biogenesis and ATP production promotes survival in *Escherichia coli*. Sci. Rep..

[35] Kim J-S (2018). DksA–DnaJ redox interactions provide a signal for the activation of bacterial RNA polymerase. Proc. Natl. Acad. Sci..

[36] Traxler MF (2008). The global, ppGpp-mediated stringent response to amino acid starvation in *Escherichia coli*. Mol. Microbiol..

[37] Durfee T, Hansen AM, Zhi H, Blattner FR, Jin DJ (2008). Transcription profiling of the stringent response in *Escherichia coli*. J. Bacteriol..

[38] Olvera L (2009). Transcription analysis of central metabolism genes in *Escherichia coli.* Possible roles of σ38 in their expression, as a response to carbon limitation. PLoS ONE.

[39] Xavier KB, Bassler BL (2005). Regulation of uptake and processing of the quorum-sensing autoinducer AI-2 in *Escherichia coli*. J. Bacteriol..

[40] Oh K, Cho S-H (2014). Interaction between the quorum sensing and stringent response regulation systems in the enterohemorrhagic *Escherichia coli* O157:H7 EDL933 strain. J. Microbiol. Biotechnol..

[41] Burgess, R. R. *Encyclopedia of Genetics* (eds. Brenner, S. & Miller, J.H.). 1831–1834. (Academic Press, 2001).

[42] Gomez-Gomez JM, Amils R (2014). Crowning: A novel *Escherichia coli* colonizing behaviour generating a self-organized corona. BMC Res. Notes.

[43] Vichi J (2021). High-throughput transcriptome sequencing and comparative analysis of *Escherichia coli* and *Schizosaccharomyces pombe* in respiratory and fermentative growth. PLoS ONE.

[44] Zhao K, Liu M, Burgess RR (2007). Adaptation in bacterial flagellar and motility systems: From regulon members to ‘foraging’-like behavior in *E. coli*. Nucleic Acids Res..

[45] Liu M (2005). Global transcriptional programs reveal a carbon source foraging strategy by *Escherichia coli*. J. Biol. Chem..

[46] Chambers JR, Sauer K (2013). Small RNAs and their role in biofilm formation. Trends Microbiol..

[47] Thomason MK, Fontaine F, De Lay N, Storz G (2012). A small RNA that regulates motility and biofilm formation in response to changes in nutrient availability in *Escherichia coli*. Mol. Microbiol..

[48] Sheikh J (2002). A novel dispersin protein in enteroaggregative *Escherichia coli*. J. Clin. Invest..

[49] Rossi E, Paroni M, Landini P (2018). Biofilm and motility in response to environmental and host-related signals in Gram negative opportunistic pathogens. J. Appl. Microbiol..

[50] Lewin CS, Amyes SG (1991). The role of the SOS response in bacteria exposed to zidovudine or trimethoprim. J. Med. Microbiol..

[51] Thi TD (2011). Effect of recA inactivation on mutagenesis of *Escherichia coli* exposed to sublethal concentrations of antimicrobials. J. Antimicrob. Chemother..

[52] Podlesek Z, ŽgurBertok D (2020). The DNA damage inducible SOS response is a key player in the generation of bacterial persister cells and population wide tolerance. Front. Microbiol..

[53] Vianney A (2005). Escherichia coli tol and rcs genes participate in the complex network affecting curli synthesis. Microbiology (Reading).

[54] Ogasawara H, Yamamoto K, Ishihama A (2010). Regulatory role of MlrA in transcription activation of csgD, the master regulator of biofilm formation in *Escherichia coli*. FEMS Microbiol. Lett..

[55] Barnhart MM, Chapman MR (2006). Curli biogenesis and function. Annu. Rev. Microbiol..

[56] Ravindra Kumar S, Imlay JA (2013). How *Escherichia coli* tolerates profuse hydrogen peroxide formation by a catabolic pathway. J. Bacteriol..

[57] Baez A, Shiloach J (2013). *Escherichia coli* avoids high dissolved oxygen stress by activation of SoxRS and manganese-superoxide dismutase. Microb. Cell Fact..

[58] Zheng M (2001). DNA microarray-mediated transcriptional profiling of the *Escherichia coli* response to hydrogen peroxide. J. Bacteriol..

[59] Djaman O, Outten FW, Imlay JA (2004). Repair of oxidized iron-sulfur clusters in *Escherichia coli*. J. Biol. Chem..

[60] Jang S, Imlay JA (2010). Hydrogen peroxide inactivates the *Escherichia coli* Isc iron-sulphur assembly system, and OxyR induces the Suf system to compensate. Mol. Microbiol..

