# Author Correction: Transcriptomic profiling of *Escherichia coli* K-12 in response to a compendium of stressors

**DOI:** 10.1038/s41598-022-21625-2

**Published:** 2022-10-07

**Authors:** Rama P. Bhatia, Hande A. Kirit, Alexander V. Predeus, Jonathan P. Bollback

**Affiliations:** 1 Institute of Infection, Veterinary, and Ecological Sciences, University of Liverpool, Liverpool, Merseyside UK; 2 Laboratories of Molecular Anthropology and Microbiome Research, Stephenson Research and Technology Center, University of Oklahoma, Norman, OK USA

Correction to: *Scientific Reports* 10.1038/s41598-022-12463-3, published online 24 May 2022

In the original version of this Article, the transcript abundance table was omitted from the Supplementary Information section.

The Supplementary Information file now accompanies the original Article.

## Supplementary Information

Supplementary Table 1.

